# Supplementary material for: Learning curve and surgical outcome of robotic assisted colorectal surgery with ERAS program
Source: Sci Rep. 2022 Nov 29;12:20566. doi: 10.1038/s41598-022-24665-w (PMC9709162; doi:10.1038/s41598-022-24665-w)
Supplement: Supplementary file 1 — Supplementary Information 1. [file 41598_2022_24665_MOESM1_ESM.zip › LSC Raw data-Ñ[▒K/131/P_ñw╝╨░O▒Kñσ.pdf]

[redacted]

Pathologic diagnosis:
Ascending colon, terminal ileum, and appendix, right hemicolectomy
--- Diverticulosis with chronic follicular diverticulitis. The appendix, ileocecal valve, bilateral cut ends and mesocolon are unremarkable.

Gross description:
The specimen consists of a segment of colon, 7.5 cm in length, and 6.5 cm in distal cut end circumference, and a segment of terminal ileum, 6 cm in length, and 3.5 cm in cut end circumference. An unremarkable appendix, 5 cm, is noted. There are three diverticula, up to 0.5 cm, noted at the mucosa of ascending colon, which are 1.8 cm from the ileocecal valve. The appendix, ileocecal valve, and both cut ends are free of tumor. Representative part for section: A) proximal cut end B) distal cut end C) appendic D) ileocecal valve E-G) lesions.

#T-59420_2 #M-32700_2 0 1105 000000
#T-59420_2 #M-43000_2 0 1105 000000
#T-59200_2 #M-09183_2 0 1105 000000

已電子簽章

敬告：本單僅供醫師參考使用，不具任何申請效力！！
請病患妥為保管，若不慎遺失需自負個人資料外洩責任！
